# Aligning Best Practices: A Guiding Framework as a Valuable Tool for Public Health Workforce Development with the Example of Ukraine

**DOI:** 10.3390/ijerph18179246

**Published:** 2021-09-01

**Authors:** Olga Gershuni, Katarzyna Czabanowska, Genc Burazeri, Vesna Bjegovic-Mikanovic, Grzegorz Juszczyk, Anna Cichowska Myrup, Volodymyr Kurpita

**Affiliations:** 1 Department of International Health, Care and Public Health Research Institute–CAPHRI, Faculty of Health, Medicine and Life Sciences, Maastricht University, 6229 GT Maastricht, The Netherlands; kasia.czabanowska@maastrichtuniversity.nl (K.C.); genc.burazeri@maastrichtuniversity.nl (G.B.); 2 School of People & Healthcare Studies, Fontys University of Applied Sciences, 5631 BN Eindhoven, The Netherlands; 3 Institute of Public Health, Faculty of Health Sciences, Jagiellonian University, 31-126 Krakow, Poland; 4 Department of Public Health, Faculty of Medicine, University of Medicine, 1005 Tirana, Albania; 5 Faculty of Medicine, Centre School of Public Health and Management, University of Belgrade, 11000 Belgrade, Serbia; bjegov@med.bg.ac.rs; 6 Department of Public Health, Medical University of Warsaw, 02-091 Warsaw, Poland; gjuszczyk@pzh.gov.pl; 7 Division of Health Systems and Public Health, Public Health Services, WHO Regional Office for Europe, 2100 Copenhagen, Denmark; cichowskaa@who.int; 8 Center for Public Health of the Ministry of Health of Ukraine, 04071 Kyiv, Ukraine; vladkurpita@gmail.com

**Keywords:** public health, public health workforce, public health workforce development, scoping review, policy analysis, framework

## Abstract

*Background:* At present, in Ukraine, there is an insufficient capacity for up-to-date surveillance of the health status of the general population; public health (PH) promotion and disease prevention activities are scarce. Additionally, there is an urgent need to ensure, develop and support an efficient public health workforce (PHW) and appropriately address existing health issues. Ukraine currently introduces PH system reforms in line with its current burden of disease, the epidemiological profile and the Essential Public Health Services (EPHOs). This analysis aims to propose a pragmatic framework to provide guidance and recommendations related to the development, support and planning of the PHW in Ukraine. *Methods:* We constructed a framework based on a previously published scoping review and analyzed various policy analysis approaches. In line with the recommendations found in the literature and the best practices used elsewhere, this method enabled the construction of a framework for facilitating successful PHW development. In addition, an expert workshop was held, serving as a reality check for identifying crucial areas of the PH system in Ukraine. *Results:* The proposed framework includes a country’s background, the evidence and available policy options, such as the health system (including core functions, organizational resources, regulations and norms), health system capacities (including human resources; PH capacity assessment; datasets and databases; forecasting strategies; licensing, accreditation and quality assurance) and capacity building (including PH education, training, core competencies and ethical and professional codes of conduct). To facilitate and support effective implementation of the framework, we propose (1) implementing strategies to facilitate changes in attitude, behavior and practices among the citizens; (2) implementing strategies to facilitate the necessary behavioral changes in the PHW; (3) implementing strategies to facilitate the necessary organizational and institutional changes; (4) implementing strategies to facilitate system changes and (5) identification of potential barriers and obstacles for the implementation of these strategies. *Conclusion:* The report highlights the practical tactics and best practices for providing suggestions for PHW support and planning. The employment of prominent analytical tools and procedures in policymaking processes suggests an effective strategy for PHW development in Ukraine.

## 1. Introduction

While the health workforce generally enjoys much attention among researchers, far less evidence related to public health workforce (PHW) development and planning exists [1]. Currently, many challenges influence the development of the PHW worldwide, including population aging, the burden of non-communicable diseases (NCDs), health inequalities in a globalized world, migration and its related consequences for the health sector and social welfare systems [2,3,4]. All these challenges are imposed on the design of rational and evidence-based strategies for planning and developing a PHW capable of dealing effectively with public health (PH) problems. A PHW, depending on the specific country context, exhibits different qualifications, job profiles, availability and requirements for career paths and, importantly, remuneration schemes. There is a growing consensus that even between neighboring countries, the impact on labor market participation and recruitment in the PH sector is not comparable [5,6]. The situation is much more complex in those countries of the World Health Organization (WHO) European Region characterized by civil unrest and armed conflicts, including Ukraine.

### 1.1. Context in Ukraine: Current Burden of Disease, Challenges and Reforms

The life expectancy at birth in Ukraine is one of the lowest in the WHO European Region [7]. Life expectancy at birth is 72 years, which is 10 years less than in Austria, Belgium, Finland and the Netherlands [8]. Adult mortality plays a significant role, but Ukraine also exhibits a high burden of mortality related to traditional health problems, including poor maternal and child health [7]. Regarding behavioral factors, smoking and harmful alcohol consumption play a significant role in the overall Ukrainian burden of disease [7]. For example, ischemic heart disease (highly associated with smoking) leads the top 10 causes of death in Ukraine at an alarming 633.5 deaths per 100,000 people in both gender groups [9].

Based on the present epidemiological profile, the most pressing need in Ukraine is to develop a PH system that will fulfil all the Essential Public Health Operations (EPHOs) and address the current burden of disease and health system challenges [6,7]. Next to the current health care reforms, there is also an ongoing process of PH legislation development in the country [10]. The available PH program descriptions are often challenging to interpret, and clear links between the specific objectives and expected (long- or short-term) goals are not always straightforward [11]. One of the main objectives of new reforms is shifting from treatment policy to strengthening and maintaining health and preventing diseases. Building such a PH system will be one of the requirements of the Association Agreement between Ukraine and the European Union. The goal will be to create a system where every head of the central authorities and local executive authorities will consider the consequences of their decisions on PH conditions [12].

Furthermore, priority will be granted to measures that will help people avoid illness and injury. The current health reforms in Ukraine are in line with “Health 2020. A European Policy Framework supporting action across government and society for health and well-being” [13], which precedes the current WHO policy document “The European Programme of Work, 2020–2025—United Action for Better Health” [14]. Having this perspective in mind, one of the most important goals of the ongoing Ukrainian health reform is preserving the health and productivity of all citizens, similar to the approach employed in other countries of the European Region [15]. Nonetheless, PHW is lagging far behind the current challenges; hence, there is an urgent need to establish a clear and effective strategy for PHW development and planning in Ukraine.

### 1.2. Aim

This analysis proposes a pragmatic framework that can provide guidance and recommendations related to the development, support and planning of a PHW in Ukraine in line with its current burden of disease, the epidemiological profile and the EPHOs. The best evidence used for considerations can simplify efficient and adequate planning of a PHW given the variations in health contexts. Furthermore, the framework can serve as a quality improvement tool to assess the current situation and strive for capacity building. These considerations presented in the framework are based on (1) a detailed analysis of the existing PHW development plans in other countries [1] and (2) document analysis of various policy tools and steps that can be used to support the implementation of a PH development strategy [16,17].

## 2. Materials and Methods

The methodology of this analysis is a combination of several analytical methods structured in a framework: document analysis of various policy analysis approaches [16,17] in combination with a scoping review of available PHW development plans [1]. In addition, we have considered using the “fishbone” or Ishikawa diagram [18] for the factors influencing PHW development. More specifically, we aimed to construct a model based on a comprehensive Strategic Capacity Planning Framework [19] that applied the elements of PHW development strategies and linked them with policy analysis steps in the specific context and circumstances of Ukraine.

### 2.1. Document Analysis of Considerable Policy Analysis Approaches: The Steps and Implications for Examination

In this paper, we did not perform policy analysis; instead, we considered using the steps described by Collins [16] in the tool for health policy analysis through a magnifying glass of critical questions presented by Fretheim et al. [17].

Policy analysis is a complex process that “illustrates the need for interventions that highlight important policy issues, improve the policy implementation process and lead to better health outcomes” [16]. It is also a practical approach when various interests are involved in complex and multi-faceted systems. Policy analysis is considered a generic name, and it is gaining increasing prominence, although it is not considered a unified field of study yet [16,20]. Conventionally, policy analysis denotes a wide array of methods and instruments employed to evaluate the characteristics and features of established policies [16]. Many approaches available to date display the attempts to carry out the policy analysis. However, the practical aspects of policy analysis in PH in developing countries or countries facing major policy reforms are still lacking [21]. Therefore, policy analysis may constitute a valuable tool in PH to investigate highly contextualized issues and challenges [22]. The main concern of policy analysis relates to the outcomes of health policies, including the effects and impact on the population [16]. Deborah Stone [23] described policy analysis as a process that always contains a political aspect. There seems to be a gap between the various policy actors’ specific interests in the process of policymaking as such and policy implementation. Sometimes, new policies stay within the formal requirements simply because of the apprehensive attitudes of some influential policymakers [24]. Due to its intersectorality and often unclear position within the health and social care system, the PHW is seemingly a topic which yields itself to policy analysis.

The tool presented by Colins [16] simplifies and offers a step-by-step approach to evaluate healthcare related aspects. The steps (“define the context; state the problem; search for evidence; consider different policy options; project the outcomes; apply evaluative criteria; weigh the outcomes, and make the decision”) are structured and efficient when it comes to making decisions in health care [16]. Once the policy options are identified, practical steps toward its implementation can be taken. The practicality and effectiveness of any policy or policy steps can be evaluated by applying the questions clarifying implementation needs and possibilities. For example, Fretheim et al. [17] defined four entry questions for a policy to be implemented: “What are the potential barriers to the successful implementation of a new policy?”; “What strategies should be considered in planning the implementation of a new policy to facilitate the necessary behavioral changes among healthcare recipients and citizens?”; “What strategies should be considered in planning the implementation of a new policy to facilitate the necessary behavioural changes in healthcare professionals?” and “What strategies should be considered in planning the implementation of a new policy to facilitate the necessary organisational changes?” [17].

### 2.2. Scoping Review: The Objectives of Public Health Workforce Development Plans

The scoping review we used in this analysis was published in 2018, answering the central question about common traits and trends of different PHW development strategies, plans and frameworks [1]. The review was a portion of a wider project supported by the WHO Regional Office for Europe, with a focus on the PHW development strategies for Ukraine.

A scoping review is an approach that provides clarification and mapping of critical definitions [25,26,27]. In this scoping review [1], the authors sought to find a uniform process for PHW development based on the available international evidence and supporting PHW planners at different administrative levels in the European Region. Due to the complexity of the information and the diversity of the concepts, authors examined the scientific and gray literature. The combined search strategy resulted in the identification of the common nine measures. The complete set included (1) alignment between the 10 Essential Public Health Operations (EPHOs); (2) regulations and norms; (3) capacity assessment; (4) datasets and databases; (5) workforce development strategies, planning and management; (6) education, training, core competencies and models; (7) licensing, accreditation and credentialing; (8) forecasting strategies for enumerating and quotas and (9) ethical and professional codes of conduct. The authors hoped that the findings of the scoping review [1] would encourage PH organizations to use the set of proposed measures to improve and develop a PHW. The methodology and the identified measures were considered appropriate and in line with the current research aims. The complete documentation of the search strategy used in this analysis is identical to a scoping review [1].

### 2.3. Outline of the Framework

**Figure 1:**
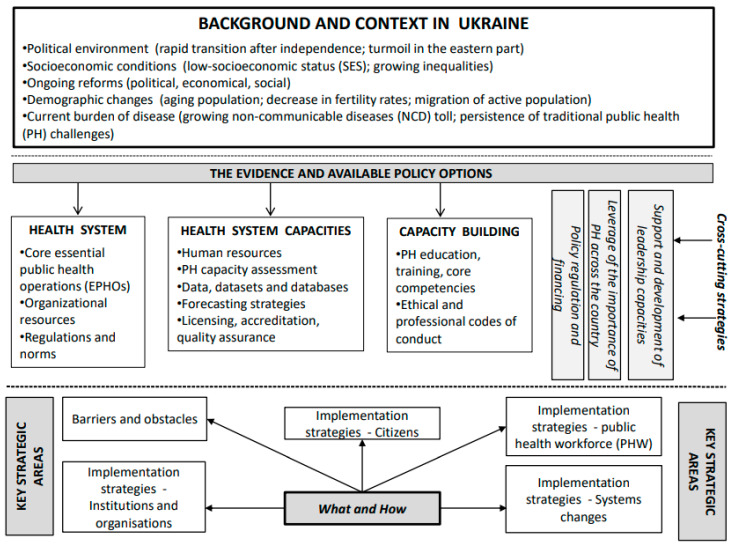
The framework of considerations for a public health workforce development plan in Ukraine. Source: adapted from Ruderman and Grason [19].

In this analysis, we concentrated on relevant evidence of policy analysis concepts and outcomes of the scoping review [1]. Blended information was then used to draft the framework (Figure 1). The Maternal and Child Health Strategic Capacity Planning Framework (CAST-5) [19] was singled out as a model that could guide the framework’s development. The research team reached a consensus on applying the CAST-5 structure due to the practicality and focus on capacity needs. In addition, theoretical approaches for policy analysis were blended [16,17,28], suggesting essential areas for the development and planning of a PHW in the context of Ukraine and for the construction of the framework.

Additionally, a workshop was conducted with the critical leadership staff of the Ukrainian Public Health Centre (UPHC). It was held in Kyiv in January 2019. The workshop served as a reality check in identifying critical areas for the improvement and development of PHW capacities.

The following policy analysis-based considerations that were employed are represented in a top-down sequence (Figure 1): (i) background and context; (ii) evidence and the available policy options; (iii) the key strategic areas for intervention and (iv) “what and how” (the implementation approach).

(i) Background and context

This is the starting point that helps to identify the country context and country profile. The societal values, political situation and structural changes, such as health reforms, will be carefully investigated concerning direct population risks. The prominent characteristics should be mapped to understand context-related problems [16].

(ii) The evidence: available policy options

After a problem or burden is identified, evidence collection can take place [16]. An examination of the published sources was adopted in order to propose a set of considerations for the PHW development strategy. Based on a scoping review, several essential measurements for the development of the PHW were selected for content presentation [1]. The identified evidence and policy options have been grouped under the three headings offered by the CAST-5 [19]. These are the health system (including core functions, organizational resources, regulations and norms), health system capacities (including human resources; PH capacity assessment; data, datasets and databases; forecasting strategies; licensing, accreditation and quality assurance), and capacity building (including PH education, training, core competencies and ethical and professional codes of conduct).

(iii) The key strategic areas of implementation

According to practical support tools of evidence-informed policymaking in the health field [17], the challenge is to materialize and implement the policy in practice after making a policy decision. Such a decision action choice can be made according to a best-fit scenario (whether being a policymaker or supporting policymakers in their decision making) and appropriate and potentially helpful implementation programs [17,28]. As suggested by the identified policy tool [17], five key strategic areas can be considered to facilitate the potential implementation of the recommended PHW development plan in Ukraine. These areas include (i) barriers and obstacles; (ii) strategies for facilitating the necessary changes among Ukrainian citizens; (iii) strategies for facilitating the necessary changes in a PHW; (iv) strategies for facilitating the necessary organizational and institutional changes and (v) strategies for facilitating the necessary systems’ changes.

(iv) What and how

A detailed analysis of “what” and “how” was performed using the the “fishbone” or Ishikawa diagram [18] to identify the factors influencing PHW development. The results of this analysis, recommendations found in the literature and best practices used elsewhere [2,18,28,29,30,31,32,33,34,35,36] contributed to the construction of the framework of considerations for facilitating a PHW development plan in Ukraine (Figure 1). We assume that the methods and concepts based on the analyzed data we used for the content are comparable to the situation in Ukraine and represent the challenging aspects of reforms in countries developing their health systems.

### 2.4. Expert Workshop

The workshop was designed to identify the problems and needs concerning the Ukrainian Public Health Centre (UPHC) assessment, leading to the subsequent improvement of its capacities and workforce development. It took place on 30 and 31 January 2019 and facilitated open conversations applying the Art of Hosting methodology, resulting in a highly participatory and co-creative process of innovation to address complex challenges [37]. The participants consisted of 10 senior staff members from the UPHC (including the director general and the first deputy director), 3 staff members from 2 different regional public health centers and 1 consultant from the Ministry of Health, as well as 2 senior representatives of the Norwegian Public Health Institute and representatives of the WHO Country Office, WHO EURO and the Association of the Schools of Public Health in the European Region (ASPHER). Stakeholder engagement and ways of working together were the important strategies employed during the workshop. The participants carried out discussions in small groups based on guiding questions related to the assessment of current capacities, needs for improvement and potential barriers to the implementation of new strategies. The reports from the groups were summarized, synthesized and analyzed based on the content.

## 3. Results

### 3.1. Results According to the Framework Steps

The framework of considerations for the development of a PHW in Ukraine (Figure 1) was designed based on the CAST-5 visual tool [19]. The framework is adjustable to the country-specific context and statutory and environmental circumstances. It should also include three key vertical areas: financing and effective policies, leveraging the importance of PH across the country and leadership. The government, PH organizations and the PHW themselves are responsible for raising the awareness of those who will implement the change (Figure 1).

As identified in the construction of the framework (Figure 1), five areas were applied to the Ukrainian context as an example of how the framework could be used in the specific context. A pragmatic framework supported guidance and recommendations related to the development and planning of a PHW in Ukraine in line with its current burden of disease, the epidemiological profile and the EPHOs.

(i) Background and context

To present the context, we considered mainly the Ukrainian Ministry of Health (MoH) website [38] and WHO Health for All database [39] due to clear presentation of demographical aspects, health statuses, risk factors and up-to-date statistics. Other related publications, including recently published papers revealing the Ukrainian context [10,40,41], were acknowledged. The overall context in Ukraine should be comprehensively considered, including the following aspects: (1) the political situation (in light of the ongoing turmoil in the eastern part of the country); (2) the socioeconomic situation; (3) the ongoing reforms in all sectors; (4) the demographic transition (aging population) which inevitably leads to epidemiological transition and a rapid shift toward NCDs; and (5) the (still) existing traditional PH issues including infectious diseases, particularly tuberculosis and HIV and AIDS.

(ii) Evidence and available policy options

Health System: A detailed assessment of the current health care system should be conducted. The assessment should include the current burden of disease, population health and health system performance (projections, trends and scenarios for the future) and a clear definition of the country’s primary mission, vision and goals, as well as the core functions and services concerning PH within the current Ukrainian context. Furthermore, it is essential to compile a comprehensive inventory of PH institutions and organizations, match their profile with the EPHOs and clearly define their services, functions and the needed PHW. Such a detailed assessment of the health care system, including human resources and capacities, would naturally lead to a proper and evidence-based establishment of performance and outcome measures on a macro level.

Health System Capacities: A compilation of a detailed inventory of the existing resources and capacities in the field of public health (e.g., population of the PHW, their distribution, age profile, gender and quality or level of training, expertise or professionalism) and identification of the gap between the available and the necessary or required resources and capacities in the field of PH are recommended.

Capacity Building: This can involve the needs assessment regarding adequate training and evaluation procedures concerning new skills and competencies for public health professionals. In addition, it describes the development or adaptation of the existing competency frameworks and development of specific strategies for capacity building. Capacity building is tailored according to the Ukrainian environment and the macro or micro level priorities.

(iii) The key strategic areas for implementation, including (iv) “what and how” analysis

The organization responsible for implementing the recommended PHW plan in Ukraine needs to consider five key areas (indicated below) to ensure effective implementation. Furthermore, to address the factors influencing PHW development, “what” and “how” aspects should be considered (Table 1). Based on the workshop results (Box 1), several capacity development-based problems and needs were specified. The questions (e.g., “what do we do, to what end and what do we need to accomplish?”) triggered a detailed approach of five key strategic areas as presented in Table 1.

### 3.2. Implementation: Problems and Needs Based on the Workshop in Kyiv

The highly participatory process proved to benefit the senior leadership of the PH system in Ukraine. It was acknowledged as a significant facilitator for holding complex conversations while converging these conversations into valuable outcomes. The results of the workshop (Box 1) significantly support the recognition of the main intervention areas for the development of PHW capacities in Ukraine.

Box 1 **Problems and needs concerning the capacity development of the Ukrainian Public Health Centre (UPHC) in Kyiv, Ukraine, based on the workshop.**

**Main problems identified:**

(1)It seemed that there was a need to redefine the mission, strategy, core services and activities closely related to them. The leadership of the UPHC would like to make sure everybody knows “What do we do?”, “To what end?” and “What do we need to accomplish the end goal(s)?”(2)It was not clear whether the UPHC delivered the services which align with its strategy and mission.(3)It seemed unclear what responsibilities were required from the UPHC and its personnel by different governance structures (including the Ministry of Health (MoH), regional health departments and local authorities), and accurate descriptions of the competencies are lacking.(4)Lack of proper communication between different governance levels. It seems that there is no explicit protocol and communication strategy concerning who, what and how. As a result, there is insufficient understanding of the needs and expectations of various actors and stakeholders.(5)The health communication and knowledge translation to society, including essential health messages (e.g., immunization), seemed ineffective or insufficient.(6)Identified problems were also related to data collection modalities, data linking, interpretation and presentation.(7)Lack of clear performance benchmarks (e.g., what can be our goal and what we should accomplish in each of these areas).(8)The participants repeatedly stressed the importance of using the PH competency framework to help assess and identify their strengths, needs and deficits.

**Needs concerning organizational self-assessment:**
Assess the core PH services that describe UPHC activities and roles within broad conditions of the health care environment;Assess organizational resources required to deliver and fulfil diverse program objectives on the system level to achieve the strategy;Perform gap analysis based on the inventory of the current capacities and a strategic business plan to follow.
**Needs concerning the development of organizational capacity**:Assess workforce needs in terms of PH core competencies;Revise the existing job descriptions to reflect better the skills of the current workforce and better plan any needed CPD;Develop a recruitment plan to address some of the identified gaps;Develop succession planning and a retention strategy.

## 4. Discussion

In the current analysis, several policy analysis-based steps combined with a scoping review were used to develop a framework that could be recommended as a potentially useful strategy to identify the areas and fundamental steps to establish a PHW development plan in the Ukrainian context. More specifically, our proposed framework suggests considering the specificity of the Ukrainian health system, its capacities and capacity building, PH policies and finances supporting PHW development. It stresses the importance of PH throughout the country by leadership and effective oversight of operations performance. However, the framework cannot thoroughly do “justice” to the complexity of the diverse processes, actors and institutional conditions that affect PHW development. Instead, it should be considered a “guiding light” to operationalize a possible assessment, leading to quality improvement of the PHW. As such, policy analysis was not performed; instead, the analysis attempted to show “what and how” could be done and the means of overcoming the barriers based on the solutions proposed by policy analysis tools. In line with the study by Collins [16], we also recommended a practical tool to improve PH outcomes and guide PHW development plans in different countries of the European Region.

While the research of El-Jardali et al. [29] focused on the retrospective perception to develop suitable policy, we employed a more prospective approach considering upcoming strategies and plans for PHW development in different contexts and circumstances, depending on the specific needs and available resources in each country. It is worth noting that the challenges and needs pointed out by participants of the workshop in the UPHC in Kyiv, Ukraine represent typical policy analysis-related issues and fit within the framework for the considerations proposed by this analysis. The ongoing PH reform is a complex process that started in 2016 and included many actors. The modern history of independent Ukraine knows many political and system changes, supporters and critics. Often, by knowing what to do, too many options exist on how to go about it. Given the particular context, not every successful policy developed in a high-income country can have measurable benefits and provide needed support for PHW development.

Even though policy analysis is not a new term, and the approach is widely used worldwide, we are still facing the challenge of “applying” a high-income country’s structure in a less well-developed country context [21,29,30,31]. Various methodologies serve as practical examples of such analysis to emphasize the interaction between and within the key players at the regional, national and international levels [16,21,22,30]. Conversely, the key players are often neglected [30], which may put the country under pressure or even cause it to fail [31]. An important point raised by Sabi and Rieker [42] highlights the engagement of civil society in health policymaking. The country authorities in South Africa supported the Treatment Action Campaign, an organization active at the national, community and provincial levels. The combination of legal actions and governmental power (constitutional law) resulted in well-grounded activities toward sustainable HIV and AIDS policy against drugs [42].

It is essential to understand the outcomes to be achieved by introducing the policy developed in other circumstances, which are not necessarily comparable to this particular country’s context. The context can be brought to light through a case study, helping to imbed the “foreign” methodology into a rather complex environment [21,29].

Various methodologies, such as the health policy triangle by Walt and Gilson [30], a tool for policymakers which was partially introduced in this study, and a simple tool for policymakers by Collins [16] share the same features. However, cautious interpretation and a tailored proposal are needed to fit into the current health system without significant interference.

All factors mentioned above are recommended to be analyzed in the Ukrainian context to ensure effective implementation of the new policy, which is the introduction of a suitable PHW development plan in this country. However, to assure success, prioritization is needed.

### Limitations

There are several limitations of the current analysis. First, a typical problem involved with this type of process is that decision makers often ignore various types of policy analysis [16,32]. Furthermore, decisions are usually made in a group setting rather than by individuals [29,33]. Additionally, a policy analysis report is only one source of information that decision makers rely upon, whereas many other competing values influence decision makers in their pressing work [34]. More importantly, different stakeholders are involved with various types of policy analysis. In our analysis, we examined several policy analysis approaches without conducting policy analysis ourselves. We suggest undertaking a policy analysis to further develop a PHW strategy in Ukraine. Therefore, decision makers and politicians in Ukraine may be confronted with competing studies based on similar or other data and presenting them with contrasting assumptions, alternatives, criteria and conclusions [33,35,36].

Of note, policy analysis always focuses on details, practical aspects and interactions between them [16,17]. Therefore, it can be employed by a specific country on either the macro level (e.g., government, national or country level) or micro level of performance (e.g., within an organization or an institution). However, the requirements on a macro level should be in place (or at least in the implementation process) so the needs on the micro level can be addressed [15,16].

The analysis and workshop were conducted in 2019, and therefore the impact of the COVID-19 pandemic was not considered in this report. The specificity of country response and preparedness should be apprised in the application of the framework.

## 5. Conclusions

The combination of eminent policy analysis tools, extensive evidence used for policymaking processes and strategic planning steps suggest an effective approach to formulate recommendations for the development and implementation of a PHW in Ukraine. In addition, this analysis underlines the distinctive strategies and best practices for the provision of essential PH services.

## Figures and Tables

**Table 1:** Key strategic areas for implementation including “what and how”.

The Key Strategic Areas for Implementation (i–v)	What	How
(i) The potential barriers and obstacles for the implementation of the proposed plan for public health workforce (PHW) development	The attitudes, behaviors and practices of the population in general may pose potential obstacles or barriers to the effective implementation of a new policy. For example, the potential obstacles regarding public health (PH) professionals may include (but are not limited to) their lack of knowledge about what is understood by PH in general and who represents PHW; lack of agreement; lack of awareness or familiarity with this issue; lack of motivation and outcome expectancy. Additionally, the barriers and obstacles regarding organizational change may include resistance and scepticism of the relevant institutions or organizations, lack of accountability, leadership, inadequate system structures and the workforce.	A mixed-method approach can be used to perform the so-called “diagnostic analysis” to assess the perceived obstacles and barriers for implementing the upcoming PHW development plan. These can be addressed by focus group discussions, in-depth interviews, interviews with key informants or stakeholders or other qualitative methods.
(ii) Implementation strategies to facilitate the attitude, behavior and practice changes among Ukrainian citizens	The aim will be to assess the perceptions of the Ukrainian population regarding the recommended PHW development plan. Thus, the general population and current PHW should be aware of ongoing PH reforms, focusing on developing a sufficient human resources plan. Moreover, it will offer assistance and raise mindfulness among the population about PH, the PHW and a profession that may be interesting to pursue for young generations.	One (or several) techniques may be appropriate, including community asset inventory, stakeholder discussions, key informant interviews and online partner surveys. A short online survey, for example, may reveal population perspectives concerning public health reforms and will be available through the webpage of the Ministry of Health.
(iii) Implementation strategies to facilitate the necessary behavioral changes in the PHW	Endorsement of the recommended framework for PHW development requires behavioral changes of PH specialists and other health professionals engaged in implementing a new policy. It might be positive if each regional PH agency will determine the appropriate method for sharing this information based on local needs (including the provision of training, workshops, etc.). However, media campaigns developed at a national level are needed to avoid differences in explaining the effects and “show how” the country can prevent contrast in delivering the results. Additionally, the media campaigns can display the country’s performance of the new PH system in general. In any case, PH is still not a well-understood concept or broadly accepted notion in Ukraine.	We recommend developing comprehensive media campaigns to increase the awareness of health professionals, other relevant stakeholders and the general population. Additionally, competency-based training and implementation of a suitable continuous professional development plan for improving and retraining the PHW might be helpful.
(iv) Implementation strategies to facilitate the necessary organizational and institutional changes	To tackle resistance to and scepticism of the relevant institutions and organizations, inadequate leadership, workforce, accountability or inadequate systems and structures, a detailed inventory of PH institutions should be in place. Such an inventory can be a systematic process with a clear mission and goals which examines the ability of institutions to provide PH services and operations. Furthermore, each institution needs to describe the objectives, programming, staffing and available resources to meet core services and priority areas. Additionally, the managers need to be fully trained to present and discuss (using comprehensive tools) the proposed plan to a team in a transformative way.	We recommend an inventory of the institutions that provide PH services and operations in Ukraine, defining their mission, strategy and objectives with the PHW to stimulate ownership and adequate participation in the process. First-line managers should be trained, giving them appropriate tools to work with their teams in a transformative and innovative way (such as developing leadership capacity). Additionally, it may be helpful to introduce training based on the models of change; training on how to deal with resistance to change and diffusion of innovation and training in political leadership, stakeholder mapping and analysis dealing with power and distance.
(v) Strategies for implementation to facilitate the necessary system changes	The suggested framework of the PHW development plan will require some changes at the general level of the health care system in Ukraine. Such modifications may include governance structure, financial issues, the delivery of health services and the induction of acceptable quality and quantity enumeration of the PHW. Furthermore, the development of adequate expertise and professionalism should be based on evidence-based interventions. Therefore, the new strategy will require some changes at the general level of the health care system in Ukraine, including governance structure, financial issues and the delivery of health services.	Assessment of the perceptions of the Ukrainian population regarding the recommended PHW development plan is necessary. We recommend the performance of gap analysis (demand–supply) between the desired and available PHW.

Source: adapted from the “fishbone” or Ishikawa diagram [18] and Fretheim et al. [17].
